# Supplementary material for: “It Makes You Feel That You Are There”: Exploring the Acceptability of Virtual Reality Nature Environments for People with Memory Loss
Source: Geriatrics (Basel). 2021 Mar 12;6(1):27. doi: 10.3390/geriatrics6010027 (PMC8005970; doi:10.3390/geriatrics6010027)
Supplement: Supplementary file 1 [file geriatrics-06-00027-s001.pdf]

Framework Analysis

Chart View of Data Matrix for Theme 1

Theme 1: Responses to Nature Scenes ('in the moment' and interview data)

**Participants**	**1.1. Engagement during VR**	**1.2 Descriptions**	**1.3 Features particularly liked**	**1.4 Familiarity of scenes**	**1.5 Triggered memories**
**01-C** **Gender:** Woman (husband with memory loss)	Described it as 'lovely', wondered if it was Porthcurno beach. Noted that children were on the beach playing and liked the cliffs and the sea. Recognised Minack Theatre.	Commented on the lovely colours.	*I've enjoyed looking at them. They're lovely pictures and that. All the colours are beautiful.*	N/D	N/D
**02-C** **Gender:** Woman (husband with memory loss)	Commented on the sea, 'incredible sand'. That was why she came back to Cornwall. Wished that she could recognise the beaches. Makes it look so good that she wanted to go into the sea. Recognised Minack Theatre. Described it as 'absolutely amazing'.	N/D	Highlighted the scenery. *I just adore the scenery. I came back from Canada for the English countryside. We were living in Toronto and had no sea. People saw the lakes and thought that was the sea so I missed it. I'm an island person. The difference is enormous, you just don't realise. So yes, the sea.*	N/D	N/D

Participants	1.1. Engagement during VR	1.2 Descriptions	1.3 Features particularly liked	1.4 Familiarity of scenes	1.5 Triggered memories
	Wow. It's incredible how you can see all around.				
**03-C (same person as 02-C) & 03-ML Gender: Woman and Man**	Noted that it looked like the Cornish coast. Felt that he should recognise it but couldn't say where it was. Said he was going underwater but didn't know the names of the fishes. Back on shore he noted that there was good climbing – he used to be a mountaineer - and wondered if it was the Mediterranean.	N/D	Loved the views.	N/D	Used to be mountaineer – went climbing in Scotland and Switzerland. Thought he had walked on the Cornish coast.
**04-V Gender:** Woman	Noted that she had forgotten how good the beach was at Porthcurno. Found the aquarium very entertaining and the technology 'remarkable'.	N/D	N/D	N/D	N/D

Participants	1.1. Engagement during VR	1.2 Descriptions	1.3 Features particularly liked	1.4 Familiarity of scenes	1.5 Triggered memories
	Recognised Minack Theatre.				
**05-ML**<br>**Gender:** Woman	Described the headset as 'a bit funny'. Described it as 'pretty' and liked the colours. Thought that the beach was like Polzeath, described the busy beach and seemed to like that. Then she had enough – 1.5 mins of video.	Said it was very good.	*It's nice to look through 'cause it's got different pla…pictures, scenery…that's what I like about that thing, I like the scenery areas. I don't like built up areas, I like scenery, beaches an' that 'cause you got fresh air an' that lot, and when it's hot it's lovely…you can sit outside and look out at people [inaudible]…that's what I like.*	N/D	N/D
**06-V**<br>**Gender:** Woman	Described it as wonderful, lovely beach – asked which beach it was. Commented on the wonders to be found underwater – 'hidden treasures'. Wondered how you could get down to the beach. Recognised Minack Theatre.	Said it was very relaxing.	N/D	N/D	N/D

Participants	1.1. Engagement during VR	1.2 Descriptions	1.3 Features particularly liked	1.4 Familiarity of scenes	1.5 Triggered memories
**07-C & 07-ML**<br>**Gender:** Woman (daughter with mother)	Daughter encouraged mother – she removed the headset after 40 secs. And then saw a little bit at the end – Minack Theatre.	Daughter thought her Mum had liked the beach part.	Thought the pictures were very good and clear.	*I think somewhere even if you haven't been, you can see, look out, maybe somewhere you've never been before and you can see , 'oh that's what it's like'. Because I've never been to the Minack Theatre. So yeah it was quite interesting* (07-C)	N/D
**08-C & 08-ML**<br>**Gender:** Woman (husband with memory loss)	He removed the headset a couple of times – after the sea at the beginning; saw the fish and then removed the headset – ended at that point. Possibly only saw snippets	Said it was 'beautiful' and 'lovely'	*The beach and the water coming in and the fish, was lovely* (08-ML)	N/D	N/D
**09-V**<br>**Gender:** Man	No commentary during the VR experience.	Used words like 'beautiful' and 'very nice'. Found it relaxing.	Particularly liked the underwater scenes and fishes - he likes visiting aquariums and finds it relaxing.	N/D	N/D

Participants	1.1. Engagement during VR	1.2 Descriptions	1.3 Features particularly liked	1.4 Familiarity of scenes	1.5 Triggered memories
**10-C & 10-ML** **Gender:** Woman (husband with memory loss)	***I'm on the beach****. Haven't been on the beach in ages! Ooh, it's lovely. Which beach is it, maid?...Oh now I'm under the sea! Look at all these fishes. It's good innit? Wow!...and now there's someone comin' in on a boat. Hello! Ahhh. The waves are lovely*. (10-ML)	Relaxing – may refer specifically to the sunset. Later on in interview he said he found it 'calming'.	Particularly liked the fishes (his wife added that he likes nature documentaries)	N/D	Used to go to the seaside on holidays every year. Beautiful beaches in Devon. *Used to drive up with the children, everyone in the car, buckets and spades, the lot! Teignmouth usually. Sometimes Dawlish.* (10-C)
**11-C** **Gender:** Man	Nice beach, wondered where it was. Wanted to know where the underwater footage was from. Recognised Minack Theatre.	Described it as lovely.	N/D	N/D	N/D
**12-ML** **Gender:** Woman	Enquired which beach it was. Found it strange, described the figures as having red edges. Saw a huge figure come out of the sea. Described the fish as beautiful. Didn't like it when she found	Described it as 'pretty amazing' and 'soothing'.	Sea was beautiful, she liked water, found the colours beautiful, liked watching fish.	N/D	N/D

**Participants**	**1.1. Engagement during VR**	**1.2 Descriptions**	**1.3 Features particularly liked**	**1.4 Familiarity of scenes**	**1.5 Triggered memories**
	herself sitting on a rock.				
**13-ML**<br>**Gender:** Woman	*Oh, now I'm in the sea with the fish!... Back on the beach. Who's that over there?*	She said it made her feel nice - *Tis calming isn't it, seeing the waves?*	Liked the beach the best. Loves the coast because she grew up in Crantock, near Newquay. Did not go there much - *Occasionally my daughter takes me up to Crackington. But not often. They got lives, you know*	Recognised the Minack Theatre, had gone there a long time ago.<br>*13-ML: Oh I recognise this. Is it the er…the?*<br>*R: Is it the Minack?*<br>*13-ML: Minack Theatre that's it! I went once, oh, long time ago.*	*…spent many a summer holiday in Crantock. My Mother was still there, see. And we took the kids, and then the grandkids when they come along*<br>She said she missed the coast terribly – *it's part of me*
**14-V**<br>**Gender:** Woman	*It feels weird going all 'round! Oh I've got some fish*	N/D	N/D	N/D	N/D
**15-V**<br>**Gender:** Woman	Asked which beach it was and thought it was 'lovely' to see under the sea.	N/D	Loved the fish – *I admire all those people that can swim underwater and see all the things.* (She had never learned to swim)	N/D	Brings memories back, going to the seaside. She had not been to the beach for years; she walks along the cliff but not the beach.

**16-C & 16-ML** **Gender:** Woman (husband with memory loss)	Carer described it as 'beautiful', 'lovely colours'. She enjoyed it and informed her husband what was happening. *Oh that's under the sea, oh that's looking, oh that's beautiful! Absolutely! Oh we like these sort of things. [husband's name], you'll enjoy this one. The coral, yeah, it's lovely [husband's name]. You'll enjoy that. Yeah, all the lovely colours. Yeah, the fish are in there. It's lovely. So long as I don't see a shark, I shall be alright. [laughs]*<br><br>*The water coming up on me feet.* (16-ML) Recognised the Minack Theatre. Took the headset off but was encouraged to keep it on.	N/D	*…we like that coral one. Where you're under the sea. Because we watch those programmes anyhow* (16-C)	He seemed to be quite excited to have seen the Minack Theatre (had never visited it) and when asked which parts he liked, he responded Minack Theatre.	He said that he had seen the corals when in Egypt. He had been in the British Army as a Rifleman in the 1950s doing his National Service - had gone down the Gulf of Suez to the rest camp and had swam in the sea – lots of little fishes and coral to be seen.

Participants	1.1. Engagement during VR	1.2 Descriptions	1.3 Features particularly liked	1.4 Familiarity of scenes	1.5 Triggered memories
**17-C & 17-ML**<br>**Gender:** Woman (mother with memory loss)	The woman with dementia thought she knew where the beach was but she hadn't been thinking about Porthcurno. Commented on the loads of fish.	N/D	*…I mean it makes you feel, good in a way, you know, just as you can see it, and um, you don't see anything else, just all those fishes, the massive fish.*	Liked familiar places but said would like new places as well.	N/D
**18-V**<br>**Gender:** Woman	*I feel I want to go for a swim. Oh that's the Minack isn't it?*	Said it was lovely. *…it was lovely 'cause it gives you, I don't know, a sense of freedom, yes… And certainly when it was just the sea, the empty sea, I'd just like to go and have a nice swim.*	N/D	N/D	*Because when you look at the beach you think of when you were there with your own family on the beach and doing different things and then you start thinking where you used to go and things like that, yeah…*cause when the children were younger we used to take a boat and go down to Christchurch and that. So you'd start talking about that, like 'remember when…' And my husband was a lifeguard so you could go back to that.

**Participants**	**1.1. Engagement during VR**	**1.2 Descriptions**	**1.3 Features particularly liked**	**1.4 Familiarity of scenes**	**1.5 Triggered memories**
**19-ML**<br>**Gender:** Woman	Enquired as to which beach it was and noted she had difficulty in accessing beaches. Had a problem with her glasses.	Said it was really nice. *…it made me feel good actually. It was, I don't know, it was, it was really like… 'oh wow, look', but you know, 'cause it was just really nice pictures and everything, to look around and look at the like… it's not just a picture, if you wanna look up on the cliff, you can look up; if you wanna look down, you can look down. I think the whole experience is really good, very good indeed.*	*…it made me feel good actually. It was, I don't know, it was, it was really like… 'oh wow, look', but you know, 'cause it was just really nice pictures and everything, to look around and look at the like… it's not just a picture, if you wanna look up on the cliff, you can look up; if you wanna look down, you can look down. I think the whole experience is really good, very good indeed.*	N/D	N/D

Chart View of Data Matrix for Theme 2

Theme 2: Responses to VR Experience

Participants	2.1 Immersion (presence)	2.2 Opportunity to 'go somewhere'	2.3 Opportunity to see and 'interact' with people	2.4 'Strange and scary'
**01-C**<br>**Gender:** Woman (husband with memory loss)	N/D	N/D	N/D	N/D
**02-C**<br>**Gender:** Woman (husband with memory loss)	N/D	N/D	N/D	N/D
**03-C (same person as 02-C) & 03-ML**<br>**Gender: Woman and Man**	N/D	N/D	N/D	N/D
**04-V**<br>**Gender:** Woman	*It's quite an amazing sort of thing. It's almost like an experience without the smells [laughs].*	N/D	N/D	N/D
**05-ML**<br>**Gender:** Woman	N/D	N/D	N/D	N/D
**06-V**	N/D	N/D	N/D	N/D

Participants	2.1 Immersion (presence)	2.2 Opportunity to 'go somewhere'	2.3 Opportunity to see and 'interact' with people	2.4 'Strange and scary'
**Gender:** Woman				
**07-C & 07-ML** **Gender:** Woman (daughter with mother)	N/D	N/D	N/D	Underwater scenes might have been confusing for the PwD.
**08-C & 08-ML** **Gender:** Woman (husband with memory loss)	N/D	N/D	N/D	N/D
**09-V** **Gender:** Man	He said that he felt as if he were there. *…I was closing me ears off to that [the background noise]. Concentrating on the panoramic views.*	N/D	N/D	N/D
**10-C & 10-ML** **Gender:** Woman (husband with memory loss)	N/D	N/D	N/D	N/D
**11-C** **Gender:** Man	*…I mean you watch the television, and er, you see it on a screen in front of you, but you also see the surrounds, of the, things in*	N/D	N/D	N/D

Participants	2.1 Immersion (presence)	2.2 Opportunity to 'go somewhere'	2.3 Opportunity to see and 'interact' with people	2.4 'Strange and scary'
	the room. But, no, with that, virtually, you're in it, aren't you? It's fantastic.			
**12-ML Gender:** Woman	N/D	N/D	N/D	*Yeah it was pretty amazing really. Except where I was perched on a cliff at the end. That took me by surprise when I looked down. Cause I would definitely not get into that position in normal life [laughs].* Said that she had a fear of heights and described how when on a hill in Scotland with a drop on each side she had to sit down and cling to the hillside.
**13-ML Gender:** Woman	N/D	N/D	N/D	N/D
**14-V Gender:** Woman	N/D	N/D	N/D	N/D
**15-V Gender:** Woman	*…It makes you feel as if you're at the sea, right at the sea, doesn't it?*	N/D	N/D	N/D

Participants	2.1 Immersion (presence)	2.2 Opportunity to 'go somewhere'	2.3 Opportunity to see and 'interact' with people	2.4 'Strange and scary'
	…it makes you feel that you are there [emphasis] on the sand, with the sea rolling in			
**16-C & 16-ML** **Gender:** Woman (husband with memory loss)	N/D	N/D	N/D	N/D
**17-C & 17-ML** **Gender:** Woman (mother with memory loss)	N/D	N/D	N/D	N/D
**18-V** **Gender:** Woman	*…it was just like being there.* *…and I've got a beautiful view where I live, so I can look out, but I'm looking through a window whereas that [the VR], you're there, which is the difference, yeah.*	N/D	*…I liked seeing the people on the beach and I liked to, playing the Frisbee thing, you know the one on the beach and he was standing there and I thought, 'well is he gonna talk to me?!' you know [laughs] and I saw the chap in the water catching it and it was just nice to be 'in' there, especially when the surfer came down by your side and went down, it was just like being there.*	N/D
**19-ML**	N/D	*…most places in Cornwall are very cliffy. There's only a few*	N/D	N/D

Participants	2.1 Immersion (presence)	2.2 Opportunity to 'go somewhere'	2.3 Opportunity to see and 'interact' with people	2.4 'Strange and scary'
**Gender:** Woman		*places you can actually get to. So yeah, I think that [the VR] is lovely, and it's really nice yeah.*<br><br>*…the going under the sea. And just being able to look around like you're walking somewhere – like you're going somewhere. It looks, it's really good, yeah.*		

Chart View of Data Matrix for Theme 3

Theme 3: Responses to VR Equipment

**Participants**	**3.1 Heaviness of equipment**	**3.2 Difficulties with body movement**	**3.3 Difficulties with focus and glasses**	**3.4 Confidence in using the equipment**	**3.5 Use of technology in daily life**
**01-C** **Gender:** Woman (husband with memory loss)	N/D	N/D	N/D	N/D	Has a mobile and a tablet – good for information. Not on Facebook etc.
**02-C** **Gender:** Woman (husband with memory loss)	N/D	*…only problem was I wanted to be able to go round 180 degrees and I couldn't. I think a wheelie chair would be a terrific advantage because I think a lot of people aren't as mobile as I am, you know.*	N/D	N/D	N/D

Participants	3.1 Heaviness of equipment	3.2 Difficulties with body movement	3.3 Difficulties with focus and glasses	3.4 Confidence in using the equipment	3.5 Use of technology in daily life
**03-C (same person as 02-C) & 03-ML Gender: Woman and Man**	N/D	N/D	N/D	*…Seemed to be, I mean you put it on and took it off. Will you always be there to put it on and take it off?* (03-ML)<br><br>His wife observed that he would not able to use the equipment himself and would need help to do whatever was necessary.	He checked with his wife on what technology he used. He said that he had telecommunications degree and should be using technology but didn't. *You used to use all these things but you don't get along with it any more. I'm the one with the computer now. And mobile phone. You don't telephone out. You barely turn on the telly unless I do it and find the channel [both laugh].* (03-C)
**04-V Gender:** Woman	N/D	N/D	N/D	N/D	N/D
**05-ML Gender:** Woman	Found the headset 'a bit heavy'	N/D	N/D	*Oh I would need someone to do it, yeah. I couldn't do that sort of thing. That's the first time I've done that, yeah.*	N/D

**06-V**<br>**Gender:**<br>Woman	N/D	N/D	N/D	N/D	Had a mobile phone and used a computer. *Yeah well I've had to learn because it's not something that came easily or obvious. I went to classes to learn about computers. But I think that…well quite a long time ago…about 15, 17 years ago, because I was volunteering with the Alzheimer's society and they said that I had to be, you know, techno, and I wasn't, so I got on with it 'cause they supplied me, in the beginning, with a computer you see, and I had to do everything through it. But I've learned a lot more since, you see, because you learn as you go along, how to attach things and how to do spreadsheets and…*you just learn it, don't you?

Participants	3.1 Heaviness of equipment	3.2 Difficulties with body movement	3.3 Difficulties with focus and glasses	3.4 Confidence in using the equipment	3.5 Use of technology in daily life
**07-C & 07-ML** **Gender:** Woman (daughter with mother)	Though the headset was 'a big intrusive thing to put on your face.' (07-C) Felt that there was a need for something more comfortable and user friendly.	N/D	N/D	N/D	N/D
**08-C & 08-ML** **Gender:** Woman (husband with memory loss)	N/D	N/D	N/D	N/D	Did not use technology such as mobile phone and computer. *Too old in the tooth for that*. (08-ML). 08-C describes them as 'old-fashioned'.
**09-V** **Gender:** Man	N/D	N/D	N/D	N/D	He said he was not computer literate but had a phone.
**10-C & 10-ML** **Gender:** Woman (husband with memory loss)	N/D	N/D	N/D	N/D	N/D

Participants	3.1 Heaviness of equipment	3.2 Difficulties with body movement	3.3 Difficulties with focus and glasses	3.4 Confidence in using the equipment	3.5 Use of technology in daily life
**11-C**<br>**Gender:** Man	Thought the headset was a little heavy and would be improved if lighter.<br>*… But I think if you were to watch it for, if you watched a film on it for half an hour, or an hour, it would become a bit heavy then.*	N/D	Said it was blurry.<br>*…perhaps I should have had it with me glasses on because it would have been a bit more focussed but…*	N/D	Had a mobile phone and ipad but didn't consider himself to be an 'expert'. But can usually find out what he wants some way or other.
**12-ML**<br>**Gender:** Woman	*…I could see that it could make you feel a bit queasy if you stayed in it for a long time. I think if it, it's made smaller and more comfortable, then it would be beneficial. I think at the moment, it would get quite heavy after a while, and I think it probably would make you feel a bit funny.*	N/D	Described a strange light – 'edges on the figures and things'.<br>*Ooh, a huge figure came out of the sea then. Yeah they've got red edges around the figures.*<br>People had looked 'not quite real' and were a 'bit too brightly coloured'.	She thought that she would be able to operate it.	N/D

Participants	3.1 Heaviness of equipment	3.2 Difficulties with body movement	3.3 Difficulties with focus and glasses	3.4 Confidence in using the equipment	3.5 Use of technology in daily life
**13-ML** **Gender:** Woman	Headset was fine for the short time but felt that it might become heavy if it was on for a longer time.	N/D	N/D	*…but I'd need someone to set it all up, see. I'm no good with all that [laughs].*	Technology did not feature in her everyday life. Her daughter had bought her a mobile phone and it was still in the box. *I got me landline, so.*
**14-V** **Gender:** Woman	N/D	N/D	She said that she had *quite a job focussing on a lot of that*. She felt that her experience was spoiled because she could not see some of the detail. *It was clearer when I went into the…um, under the sea, and the night scene, and the sunset, that was all ok. It was all the beach and the, mostly the people, you know, and there were some orange flowers, and I couldn't work out what they were. I knew they were flowers, but I was, I would kind of like to know what kind of flowers they*	She did not think that it would be too difficult as although, she did not describe herself as 'high tech', she did not feel she was illiterate.	N/D

Participants	3.1 Heaviness of equipment	3.2 Difficulties with body movement	3.3 Difficulties with focus and glasses	3.4 Confidence in using the equipment	3.5 Use of technology in daily life
			were and I couldn't. They were just kind of, blobs of orange.		
**15-V**<br>**Gender:** Woman	N/D	N/D	N/D	N/D	She had an iphone and a computer but felt that …*I'm not very good at grasping new things. Um, and that's because I think I close my mind to it, um really. If I need, I get around it, you see. if I want to do something and I don't know how to do it, I will get round it by doing things my way, to get the end result I want, which is not a good idea.*
**16-C & 16-ML**<br>**Gender:** Woman (husband with memory loss)	N/D	N/D	N/D	N/D	They do not have a computer. She occasionally uses her mobile phone.<br>*We're not interested. It isn't our era if you know what I mean, so you know. (Carer)*

Participants	3.1 Heaviness of equipment	3.2 Difficulties with body movement	3.3 Difficulties with focus and glasses	3.4 Confidence in using the equipment	3.5 Use of technology in daily life
**17-C & 17-ML** **Gender:** Woman (mother with memory loss)	N/D	N/D	N/D	N/D	She said she didn't like technology too much and carer said she used the computer sometimes.
**18-V** **Gender:** Woman	N/D	N/D	Said that it not seem to be in focus, 'just a little bit blurred'. After adjustments it wasn't so bright so she decided to remove her glasses. Said it wasn't exactly in focus. Thought she should have worn her distance glasses, would have helped with the sharpness.	N/D	She had a computer and mobile phone.
**19-ML** **Gender:** Woman	Not painful but a 'little heavy'. She thought that because of the heaviness at the front, that there needed to be a strap sat the front so that the headset fitted properly.	N/D	Thought it should have been clearer. 'It's a bit um, pixely.' She wasn't sure whether to put the headset on top of her glasses or use her reading glasses.	She felt quite confident that she could use the VR equipment - *…I think I would be. It's just pressing a couple of buttons.*	N/D

Chart View of Data Matrix for Theme 4

Theme 4: Volunteer Staff and carers' perceptions of VR for PwD and in long term care settings

**Participants**	**4.1 Alternative means to accessing nature**	**4.2 Relieving boredom**	**4.3 Trigger memories**	**4.4 Improve mood and calming**	**4. 5 Potentially confusing**	**4.6 Inability to cope with VR equipment**	**4.7 General comments**
**01-C** **Gender:** Woman (husband with memory loss)	N/D	N/D	N/D	N/D	N/D	N/D	Thought it would be a good idea for PwD and that her husband could be interested.
**02-C** **Gender:** Woman (husband with memory loss)	N/D	N/D	N/D	N/D	N/D	N/D	Thought it would be totally 'lost' on husband and that he would be confused. Would not remember it two minutes after it was over.
**03-C (same person as 02-C) & 03-ML Gender: Woman and Man**	N/D	N/D	N/D	N/D	N/D	N/D	N/D

**04-V**<br>**Gender:**<br>Woman	N/D	N/D	N/D	N/D	Thought it could be confusing - … *for some people they would find it a bit confusing, you know, and think: 'what on earth's going on here…this is a bit weird'. Um and especially those who, you know, their memory loss is so great that they don't remember one minute to the next minute…that contradict themselves all the time you know, so you ask them if they enjoy it one minute and they'd go 'yes', and then you ask them again and they'd go 'what?',*	N/D	If the scenes were local to the area they could trigger memories. Local events like Flora Day in Helston or the Royal Cornwall Show. Eden project is quite iconic, might have seen on TV, even if they hadn't visited it.<br>Believes that familiarity help PwD feel safe.

Participants	4.1 Alternative means to accessing nature	4.2 Relieving boredom	4.3 Trigger memories	4.4 Improve mood and calming	4. 5 Potentially confusing	4.6 Inability to cope with VR equipment	4.7 General comments
					you know? [laughs] and you know, they can't even remember what they look…saw. But, I think, just, experiencing it, it would be something different.		
**05-ML** **Gender:** Woman	Thought it could be good for those who couldn't be outdoors but … you gotta have fresh air as well aint you as well?	N/D	N/D	N/D	N/D	N/D	N/D
**06-V** **Gender:** Woman	N/D	N/D	N/D	Felt that when people would 'see the beach' they would be uplifted. Could distract a PwD	N/D	Felt that she could operate the VR equipment but was less sure about some of the carers.	N/D

Participants	4.1 Alternative means to accessing nature	4.2 Relieving boredom	4.3 Trigger memories	4.4 Improve mood and calming	4. 5 Potentially confusing	4.6 Inability to cope with VR equipment	4.7 General comments
				if s/he was having a 'bad day' and improve mood.			
**07-C & 07-ML** **Gender:** Woman (daughter with mother)	Thought it could be useful. …*Sort of make them feel like they've got out of the four walls for, you know, even if it's ten minutes or something.*	N/D	N/D	N/D	N/D	N/D	Doesn't think that the nature scenes need to be of familiar places. …*I think somewhere even if you haven't been, you can see, look out, maybe somewhere you've never been before and you can see , 'oh that's what it's like'. Because I've never been to the Minack Theatre.* (07-C)
**08-C & 08-ML** **Gender:** Woman (husband with memory loss)	N/D	N/D	N/D	N/D	N/D	N/D	N/D
**09-V** **Gender:** Man	N/D	Thought it would be a	Good for memories.	N/D	N/D	N/D	N/D

Participants	4.1 Alternative means to accessing nature	4.2 Relieving boredom	4.3 Trigger memories	4.4 Improve mood and calming	4. 5 Potentially confusing	4.6 Inability to cope with VR equipment	4.7 General comments
		good idea could be stimulating.					
**10-C & 10-ML** **Gender:** Woman (husband with memory loss)	N/D	N/D	Potential to trigger memories (if it could be personalised) (10-C).	Would be calming (10-ML). Help improve mood for people who were having a bad day and couldn't get out (10-C).	N/D	N/D	N/D
**11-C** **Gender:** Man	Thought that if he couldn't go outdoors at some stage in the future it would be great. *…'Cause it brings the outside back right into you, yeah.*	N/D	N/D	N/D	N/D	N/D	N/D
**12-ML**	N/D	N/D	N/D	N/D	N/D	N/D	N/D

Participants	4.1 Alternative means to accessing nature	4.2 Relieving boredom	4.3 Trigger memories	4.4 Improve mood and calming	4. 5 Potentially confusing	4.6 Inability to cope with VR equipment	4.7 General comments
**Gender:** Woman							

**Participants**	**4.1 Alternative means to accessing nature**	**4.2 Relieving boredom**	**4.3 Trigger memories**	**4.4 Improve mood and calming**	**4. 5 Potentially confusing**	**4.6 Inability to cope with VR equipment**	**4.7 General comments**
**13-ML Gender:** Woman	*Oh yeah, I think it could be very useful. Especially in homes, you know. 'Cause I'm still in my own place, well assisted livin' 'tis. 'Cause I can still get about at the moment, see. But I know I might have to go in a home at some point. And they can't always…my sister is in [home name] in [town], and she don't get out all that much, I don't think.*	N/D	N/D	N/D	Spoke about her sister and how her dementia was more advanced than hers. She felt her sister could be confused and agitated by VR.	N/D	N/D

Participants	4.1 Alternative means to accessing nature	4.2 Relieving boredom	4.3 Trigger memories	4.4 Improve mood and calming	4. 5 Potentially confusing	4.6 Inability to cope with VR equipment	4.7 General comments
**14-V**<br>**Gender:** Woman	Thought it would be very important for those who could not access the outdoors.	N/D	Felt that someone in the early stages of dementia could look at it and recognise places.<br>*…at the early stages of dementia and you couldn't get out or something, you'd sort of, watch it and think 'oh I know there' and 'I've been there', and I think that would sort of, open up a lot of memories for people.*	N/D	N/D	Thought that PwD might find it hard to cope with the headset ('quite heavy on your face') and would not understand what 'you were trying to do for them'. Thought that it might depend on the degree of agitation the PwD experienced. Wondered if PwD could cope with moving around or if they would think they had to look forward.	VR could be an opportunity for PwD to see something different.<br>*…maybe to be taken out of that world like going under the sea, that was really lovely. And that would mean something to me, because I used to scuba dive, so that would be fantastic seeing that, but it's also something that gives people an opportunity to see something that they wouldn't see.*<br><br>Thought that PwD would benefit from having a carer helping to

Participants	4.1 Alternative means to accessing nature	4.2 Relieving boredom	4.3 Trigger memories	4.4 Improve mood and calming	4. 5 Potentially confusing	4.6 Inability to cope with VR equipment	4.7 General comments
			Thought the local aspect was very important for triggering memories				facilitate the VR session.<br>*…to have somebody talking to them, saying 'is there anything over there?' or you know, 'can you see round the rocks?' or whatever, it might inspire them to go that bit further.*<br><br>Wondered if VR would be used in care homes as it would be very time-consuming.<br>*…I think the sad thing is though…whether it would actually get used, in a, in a care setting, I don't know. Because it's time, it's gonna be*

Participants	4.1 Alternative means to accessing nature	4.2 Relieving boredom	4.3 Trigger memories	4.4 Improve mood and calming	4. 5 Potentially confusing	4.6 Inability to cope with VR equipment	4.7 General comments
							time consuming isn't it, and it's one-to-one, and everything, and maybe in care settings that's quite a difficult task isn't it, time-wise. I mean somewhere like this, one of us, if we were in this kind of situation, one of us could sit with one or two different people through a session and they could have a go, and do it yes, but I do wonder how much it would get used in a care home. I think it'd be a lovely thing to have in a care home…
**15-V**	N/D	N/D	N/D	N/D	N/D	N/D	N/D

**Participants**	**4.1 Alternative means to accessing nature**	**4.2 Relieving boredom**	**4.3 Trigger memories**	**4.4 Improve mood and calming**	**4. 5 Potentially confusing**	**4.6 Inability to cope with VR equipment**	**4.7 General comments**
**Gender:** Woman							
**16-C & 16-ML Gender:** Woman (husband with memory loss)	N/D	N/D	N/D	N/D	N/D	N/D	N/D
**17-C & 17-ML Gender:** Woman (mother with memory loss)	Thought it would be a great idea for those unable to get outdoors. (17-C)	*…she gets quite bored, and so I think going out to look at something like that, would be quite nice. Whether she'd sit there for long with it on…but you don't have to do you, you can sort of sit there for five minutes worth of…*	N/D	N/D	N/D	N/D	N/D
**18-V Gender:** Woman	*…Anyone who's had to be indoors, they*	N/D	Could bring back memories.	*…You know, especially if it was a horrible*	*…but he [her husband] wouldn't have*	*…Maybe, maybe they would need someone with them*	N/D

Participants	4.1 Alternative means to accessing nature	4.2 Relieving boredom	4.3 Trigger memories	4.4 Improve mood and calming	4. 5 Potentially confusing	4.6 Inability to cope with VR equipment	4.7 General comments
	just get that feeling of being there. Thought it would be 'lovely' and 'marvellous' for those indoors.			*afternoon and you could say 'alright we'll go down to the beach, this is what it was like…*	*been able to manage on his own. And also, I don't know, in the end, he might have got really confused because sometimes watching the television, when the, er, adverts came on he used to say 'well what are they doing on the programme?'. Like when we were watching Downton Abbey and that, and the break came in, and he would say 'well they're not in it, what's happening?'*	*all the time, if they didn't, to set it up for them. 'Cause I'm just thinking of how my husband was, and he wouldn't have been able to do that. Because unfortunately, I mean, he would always put music on for us for Saturday night while we had a nice meal. And then one night he said, well, he said 'I can't get the CD to play' and I said 'well you just press that button'. You see, it had just gone out of his mind. He, well, he lost his sort of, spatial awareness,*	

Participants	4.1 Alternative means to accessing nature	4.2 Relieving boredom	4.3 Trigger memories	4.4 Improve mood and calming	4. 5 Potentially confusing	4.6 Inability to cope with VR equipment	4.7 General comments
						thing. So, yes I think, depends, depends what type of dementia they've got, I guess. But he wouldn't have been able to do that. He would have enjoyed it…	

Participants	4.1 Alternative means to accessing nature	4.2 Relieving boredom	4.3 Trigger memories	4.4 Improve mood and calming	4. 5 Potentially confusing	4.6 Inability to cope with VR equipment	4.7 General comments
**19-ML** **Gender:** Woman	*…I think people would appreciate that, sat in hospital, because it really does feel like you're there.* <br>*….'Cause I think a lot of people with my condition, fibro and chronic pain, aren't able to get out of their beds, aren't able to go places. So that [the VR], I think, definitely would help, and I think maybe would you know, relax them, and you know, just help*	N/D	N/D	N/D	N/D	N/D	The noise of activities in the memory café meant that this participant did not hear the sound in the VR experience. *…I mean, even just walking along by the river, or going to the beach, or going to the zoo, or going…anywhere, you're sometimes…blocked. So seeing like, that [the VR], like going and having a…sometimes sitting down and just maybe chilling, and maybe having sounds to go with it. So as well as having, yes, the pictures, but maybe*

Participants	4.1 Alternative means to accessing nature	4.2 Relieving boredom	4.3 Trigger memories	4.4 Improve mood and calming	4. 5 Potentially confusing	4.6 Inability to cope with VR equipment	4.7 General comments
	them, maybe get in a better mood. Because your mood swings with chronic pain, it's up and down. Like yesterday, I didn't wanna be here – didn't wanna live. Today, I'm, oh yeah, I'm getting there. I'm really sore but, you know, and that's the sort of…						*have sound as well, so you can hear the sea…so it's more of a real connection. 'Cause it's alright watching it, but it'd be nice to hear it as well. So if you go along and say you…you can always…when go along to a beach you can always hear the roar of the sea, and it's a real nice relaxing feeling.*

Chart View of Data Matrix for Theme 5

Theme 5: Preferred nature and ease of access

Participants	5.1 Preferred nature	3.2 Difficulties with accessing preferred nature
**01-C**<br>**Gender:** Woman<br>(husband with memory loss)	*Lizard. Right across, you see down the lane, Kynance there, and you walk up the steps and it's beautiful. You can't beat it. You can't beat nature… looking over to Kynance Cove and right down to the Lizard, I love it. And what's the other place I like?...I like Coverack. Yeah, that's nice. Yeah.*	N/D
**02-C**<br>**Gender:** Woman<br>(husband with memory loss)	Preferred nature is the sea.	Not easy to access as she and her husband do not have a car. Poldhu is her favourite (has a great café), go there by bus or with other people.
**03-C (same person as 02-C) & 03-ML**<br>**Gender: Woman and Man**	N/D	N/D
**04-V**<br>**Gender:** Woman	N/D	N/D
**05-ML**<br>**Gender:** Woman	N/D	N/D
**06-V**<br>**Gender:** Woman	*well I'm a country girl at heart, you know, and um, going to the beach and walking along the cliffs and that sort of thing, it's lovely…very relaxing, yeah. I've never been a good*	N/D

Participants	5.1 Preferred nature	3.2 Difficulties with accessing preferred nature
	swimmer, so, but I would like to explore under the sea really, you know, so, I've never had the opportunity<br>She particularly liked the moors and their ruggedness.	
**07-C & 07-ML**<br>**Gender:** Woman (daughter with mother)	N/D	Access to nature depends on how wheelchair friendly it is as PwD uses a wheelchair.
**08-C & 08-ML**<br>**Gender:** Woman (husband with memory loss)	Described where they like to walk when young, with lots of flowers and birds. Had a bird table and seemed to enjoy watching the birds.	Live in the country but don't do so much walking; a hill on either side of where they live and traffic were mentioned as an explanation. They used the bus to take trips into local towns e.g. Liskeard. They did not like to shop but they liked that they were recognised when they walked around the town.
**09-V**<br>**Gender:** Man	His favourite place was Crackington, near Bude because of the rock formations. …*you got the structure of the er, spheres, different layers of er, you know, sediment.*	Said he would go to nature if he had time and 'could be bothered'.
**10-C & 10-ML**<br>**Gender:** Woman (husband with memory loss)	N/D	Don't get out to nature because they no longer have a car. They said they missed it very much.<br>10-C: Oh we do, yes. Very much<br>10-: We're restricted now, you see.<br>10-C: 'Cause there aren't so many buses up our way, and we don't tend to use them in case we, we miss the last one back, you know, we'd be stuck.
**11-C**<br>**Gender:** Man	Preferred the countryside to towns and villages. Like nature and the sea.	N/D
**12-ML**	Preferred rivers and the countryside to beaches.	N/D

Participants	5.1 Preferred nature	3.2 Difficulties with accessing preferred nature
**Gender:** Woman		
**13-ML**<br>**Gender:** Woman	N/D	N/D
**14-V**<br>**Gender:** Woman	N/D	N/D
**15-V**<br>**Gender:** Woman	She did not go to the coast very often. She described herself as a 'country person'. She described how she had been brought up on a farm and visiting the seaside had not been part of her life.<br>*I lived on a farm when I was young. And the farm was up high, and we overlooked the whole of St Austell Bay, so I could see the water out there, with all this land in between. But it was something that we never…You have to realise, that I was young 70 years ago and more, and so we just didn't go to the seaside.*<br>Her relationship with nature centred around the 'land' and not the coast.	N/D
**16-C & 16-ML**<br>**Gender:** Woman (husband with memory loss)	They used to go the beach, for example, Bude.	They no longer have a car as he does not drive which meant that they do not get out and about as much as they once did. Now use the Ring and Ride bus service to take trips to towns in Cornwall.
**17-C & 17-ML**<br>**Gender:** Woman<br>(mother with memory loss)	N/D	N/D
**18-V**<br>**Gender:** Woman	N/D	*…But I haven't got my own transport, so I can't go out as much as I probably like.*

Participants	5.1 Preferred nature	3.2 Difficulties with accessing preferred nature
**19-ML**<br>**Gender:** Woman	*…I used to be able to walk everywhere, and just go out into the woods, do walking. Now, I'm so limited. Either you got, erm, you got gates locked, so if you got a mobility scooter, they expect you to climb over this, whatever it is, you know, the fencing. So you are very stuck. And, and if your mobility's really bad and you can't afford a mobility scooter…and even then, some mobility scooters won't go on, on the rough parts of, like Cornwall.*<br><br>Missed it [being able to be out and about] a lot and said she was depressed as a result.	*…very difficult. In Cornwall, there's a lot of places where you can't get to if you're disabled because they've got steps, and you just can't get down there, so you can't go into these places. So I, whenever people go, I'm stuck behind. Because abroad, they do these, um, on the beaches they do a disabled strip all the way down, so you can like, go onto the beach.*<br>'Most places in Cornwall are cliffy'- only a few places [coastal] in Cornwall that are accessible.
